# Neutralizing Antibody Response and SARS Severity

**DOI:** 10.3201/eid1111.040659

**Published:** 2005-11

**Authors:** Mei-Shang Ho, Wei-Ju Chen, Hour-Young Chen, Szu-Fong Lin, Min-Chin Wang, Jiali Di, Yen-Ta Lu, Ching-Lung Liu, Shan-Chwen Chang, Chung-Liang Chao, Chwan-Chuen King, Jeng-Min Chiou, Ih-Jen Su, Jyh-Yuan Yang

**Affiliations:** * Academia Sinica, Taipei, Taiwan; † Center for Disease Control, Taipei, Taiwan; ‡ Taipei Mackay Memorial Hospital, Taipei, Taiwan; § National Taiwan University, Taipei, Taiwan; ¶ Taipei Hospital, Taipei, Taiwan;; # National Health Research Institutes, Taipei, Taiwan

**Keywords:** SARS, neutralizing antibody, antibody decay, mortality, pathogenesis, ADE, research

## Abstract

Using the Taiwan nationwide laboratory-confirmed severe acute respiratory syndrome (SARS) database, we analyzed neutralizing antibody in relation to clinical outcomes. With a linear mixed model, neutralizing antibody titer was shown to peak between week 5 and week 8 after onset and to decline thereafter, with a half-life of 6.4 weeks. Patients with a longer illness showed a lower neutralizing antibody response than patients with a shorter illness duration (p = 0.008). When early responders were compared with most patients, who seroconverted on and after week 3 of illness, the small proportion (17.4%) of early responders (antibody detectable within 2 weeks) had a higher death rate (29.6% vs. 7.8%) (Fisher exact test, p = 0.004), had a shorter survival time of <2 weeks (Fisher exact test, p = 0.013), and were more likely to be > 60 years of age (Fisher exact test, p = 0.01). Our findings have implications for understanding the pathogenesis of SARS and for SARS vaccine research and development.

## Abstract 2

Antibody response correlates with severity of infection.

Severe acute respiratory syndrome (SARS) is a newly emerged infectious disease. Its etiologic agent is a novel coronavirus (SARS-CoV) (*1**,**2*), which can readily infect a variety of wild and laboratory animals without causing apparent clinical symptoms (*3**,**4*), making the existence of an animal reservoir possible. In humans, SARS appears with a wide clinical spectrum, ranging from self-limited pneumonia to acute respiratory distress syndrome (ARDS) and death (*5**,**6*). Anecdotally, asymptomatic infection has also been reported (*7*).

Autopsies of SARS patients have found the virus to be widespread throughout a variety of tissues and organs (*8*). During the acute phase, the virus is found in the excreta of infected persons (*9**,**10*) and is thought to be transmitted by direct contact, droplets, or contaminated environmental surfaces. Infection can be prevented largely by good hand hygiene, although some healthcare settings and communities may be prone to the aerosolization of contaminated human excreta, and in these cases, precautionary measures should be instigated accordingly (*11**,**12*). The chain of human transmission has been successfully interrupted by public health measures, but potential reintroduction of the virus from an unidentified natural reservoir remains a concern. A wealth of clinical and epidemiologic observations have emerged and contributed to the successful control of the SARS epidemic (see Peiris et al. [13] for a review). However, information on immunity and pathogenesis is insufficient to provide a comprehensive basis for specific drug or vaccine design. Nor have animal pathogenic models been established that adequately resemble the pathogenesis of SARS in humans. Without a good experimental model to study the biologic basis for human disease, the observational data collected from reported SARS case-patients, along with the associated laboratory diagnostic tests, will continue to provide essential leads in controlling a possible reemergence of SARS. To gain a better insight into the humoral responses in the context of epidemiologic and clinical settings, we analyzed the neutralizing antibody data, along with a variety of epidemiologic elements in the database.

## Material and Methods

This retrospective analysis is based on Taiwan's nationwide database on SARS cases reported from March to July 2003 to the Center for Disease Control in Taiwan (Taiwan-CDC). The criteria for reporting SARS patients evolved over time but were principally adopted from the World Health Organization, and the total reported probable SARS patients in Taiwan were 665.

### Data

The epidemiologic database contains basic demographic information (age, sex, city/county of residence); symptoms at onset; date of onset of first symptoms; date of diagnosis; dates of hospitalization, discharge, or death; results of all epidemic investigations on contact tracing; travel history; and results of laboratory tests of reverse transcription–polymerase chain reaction (RT-PCR) on SARS-CoV and other pathogens in the differential diagnosis of atypical pneumonia. The analysis of epidemiologic data has been reported previously (*14**,**15*). The detailed laboratory data taken from molecular and serologic tests of SARS-CoV infection were compiled in a separate file that could be linked to the epidemiologic data. The concordance and discordance between various serologic tests and molecular diagnostic methods of SARS have also been reported previously (*9*). The serum neutralizing antibody was measured by microtiter assay and by enzyme-linked immunosorbent assay (ELISA) (Centers for Disease Control and Prevention, Atlanta, GA, USA) as described (*9*).

### Severity of Illness

Hospitalization served the dual purposes of isolating patients and providing health care; therefore, criteria for discharging patients, i.e., being afebrile for 5 days and clinical improvement, were stringently adhered to by the clinicians as a part of public health practice. Since no antiviral drug was known to effectively shorten the clinical course of SARS, the duration of illness, defined as the number of days between onset of fever and time of discharge from the hospital, can be assumed to reflect the clinical severity of SARS manifested by the patient. To validate the consistency of the interhospital practices in patient care in relation to the severity of patients, we collected and analyzed anonymous and computerized clinical data, focusing on oxygen supplementation and respiratory therapy, on a sample of SARS patients from 3 hospitals that represented 3 healthcare accreditation levels in Taiwan: a major medical center (National Taiwan University Hospital), a regional teaching hospital (Taipei Mackay Memorial Hospital), and a district hospital (Taipei Hospital). Regardless of hospital, duration of illness correlated highly with the supplementation of oxygen, which is a good surrogate for the level of pulmonary dysfunction (p for trend <0.001) (Table 1). Thus, in our analysis, duration of illness was used as a surrogate for clinical severity among the surviving SARS patients, and death rate was also used as severity index. For the convenience of discussion, a duration of illness <2 weeks was considered mild, 2–4 weeks as intermediate, and >4 weeks as severe. A fatal case, regardless of the length of survival, was considered severe. Our data corroborate the report that SARS patients with a severe clinical course mainly had a slower and prolonged recovery (*16*).

**Table 1:** Requirement of oxygen supplement in relation to the duration of illness among SARS-CoV–infected patients*†

Outcome	Oxygen supplement		
	None or <2 L, % (n)	>2 L by mask, % (n)	By assisted ventilation, % (n)
Duration of illness (d)			
<14	100.0 (5)	0	0
15–21	100.0 (16)	0	0
22–28	86.9 (20)	13.0 (3)	0
>28	23.5 (4)	23.5 (4)	52.9 (9)
Death	0	0	100 (11)

*N = 81; SARS-CoV, severe acute respiratory syndrome–associated coronavirus. †p value for trend <0.0001.

### Statistical Analysis

Data were analyzed with SAS software (Version 8, SAS Institute Inc, Cary, NC, USA). Differences in frequencies or proportions were tested using a χ^2^ test and by risk ratios. The continuous variables, i.e., age distribution or titers of neutralizing antibody, were compared by using the Wilcoxon rank-sum nonparametric method. Multivariate logistic regression (*17*) was used to analyze factors that can affect seropositivity, including demographic information, source of infection, and duration of illness. To adjust the time effects and other covariates of interest, the relationship between antibody titer, based on logarithmic transformation of base 2 (serum dilution) and other potential factors, i.e., age, sex, infection source, and duration of illness, was quantified by linear mixed models (*18*), which took into account the correlation between repeated measurements of each study participants.

## Results

### Participants

Specimens from all patients with probable SARS in Taiwan were serologically tested for case confirmation, with a particular focus on the convalescent-phase serum specimens, as previously reported (*9*). Positive neutralizing antibody results, which correlated well with those of ELISA (Table 2), were used as the standard assay for case confirmation. Thus, 347 of 665 reported probable SARS cases were confirmed. These included cases in 126 patients whose diagnoses were based on serologic testing alone, 121 whose results were positive by both tests, and 100 patients whose diagnoses were based on the RT-PCR alone. Of these 100 diagnoses based on RT-PCR alone, 32 had convalescent-phase serum specimens that tested negative, and 68 did not have appropriate convalescent-phase serum specimens for antibody testing because of death, loss to follow-up, or inappropriate timing of serum collection (Table 3).

**Table 2:** Comparison of SARS antibody results of ELISA and neutralization assay*

ELISA†	Neutralizing antibody‡	
	Positive	Negative
Positive	207	1
Negative	10	206
Total	217	207

*SARS, severe acute respiratory syndrome; ELISA, enzyme-linked immunosorbent assay. †Reagents were supplied by Thomas Ksiazek, Centers for Disease Control and Prevention, Atlanta, GA, USA (2 reagents). ‡Using ELISA as the reference assay, the sensitivity of the neutralizing antibody (NT) test = 99.5%, the specificity of neutralizing antibody = 95.4%.

**Table 3:** Laboratory confirmation of the reported 347 SARS-CoV–infected patients by neutralizing antibody (NT) or by RT-PCR*

NT†/RT-PCR	No. (%)
Positive/positive	121 (34.6)
Positive/negative or ND‡	126 (36.7)
Negative/positive	32 (9.2)
ND‡/positive	68 (19.7)

*SARS-CoV, severe acute respiratory syndrome–associated coronavirus; RT-PCR, reverse transcription–polymerase chain reaction; ND, data not available. †Positive = NT titer of >1:16 dilution; negative = NT titer <1:16 dilution. ‡Data not available because of loss to follow-up or insufficient number of specimens (n = 8), death (n = 55), or serum collected on the 3rd week (n = 5) of fever onset.

### Seronegative Results

**Figure 1:**
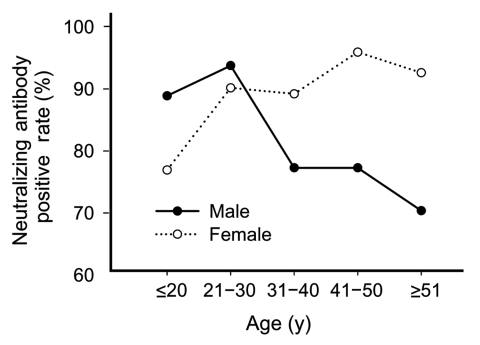
Positive rate of severe acute respiratory syndrome–associated coronavirus titer by sex and age.

We examined whether the seronegative results of the 32 patients were false-positive instances of virus detection by RT-PCR, most commonly caused by laboratory error or contamination. Cross-contamination in the laboratory should occur without any correlation with the patients' demographic or clinical parameters. The seronegative rate (19.1%, 18/94) was significantly higher in men than in women (7.5%, 14/185) (Mantel-Haenszel test, p = 0.004), but the effect of age was not statistically significant (p = 0.07 in men, χ^2^ test) (Figure 1). Based on the transmission risk of known or unknown sources, patients whose sources could not be ascertained, i.e., had no apparent history of having contact with SARS patients, were significantly more likely to be seronegative (45.1%, 23/51) than those with known sources of infection (3.7%, 8/212) (χ^2^, p<10^–7^) (Table 4). Patients with a shorter duration of illness were more likely to be seronegative; 14 (30.4%) of 46 patients with a duration of illness <14 days, 8 (9.8%) of 81 patients with illness durations of 15 and 21 days, 5 (7.8%) of 64 patients for those with 22 and 28 days, and none of those who survived for >28 days (χ^2^ for trend = 20.5, p = 0.00001). A logistic regression model confirmed that patients with a known source of infection (odds ratio [OR] = 15.6, p<0.0001) and a longer duration of illness (OR = 1.08 for each additional day of illness, p = 0.004) were more likely to possess a detectable level of neutralizing antibody than those with no discernible infection source and shorter duration of illness (Table 5).

**Table 4:** Seronegative rate of SARS-CoV patients, by source of infection*

Source of infection	Total no.	Seronegative rate, n (%)
Known		8/212 (3.7)
Hospital-associated		
Patient†	81	3/44 (6.8)
Patient's close contacts	55	1/43 (2.3)
Healthcare worker	82	2/71 (3.1)
Other worker‡	27	1/19 (4.0)
Family and social contacts	43	1/35 (2.9)
Unknown		23/51 (45.1)
Imported	22	11/20 (55.0)
Indigenous	36	12/31 (38.7)

*Seronegative rates of the 2 groups with known and unknown source of infection were significantly different (χ^2^, p<0.10^–7^). †In-hospital patients experiencing nosocomial SARS infection. ‡Including laundry workers, cleaners, clerks, and ambulance drivers.

**Table 5:** Multivariate analysis of factors affecting seropositivity and neutralizing antibody titer of severe acute respiratory syndrome (SARS) patients

Variables*†	Seropositivity*		Antibody titer†	
	OR (95% CI)	p value	Parameter estimates + SE	p value
Age (y) (n + 1 vs. n)	0.97 (0.94-1.00)	0.065	0.0056 + 0.0079	0.478
Women vs. men	1.24 (0.47-3.3)	0.67	–0.417 + 0.235	0.081
Infection source, known vs. unknown	15.6 (5.9-41.4)	<0.0001	0.248 + 0.313	0.431
Duration of illness (d) (n+1 vs. n, n = 1 through 44 d)	1.08 (1.025-1.143)	0.004	0.0638 + 0.0233	0.008
Time of convalescent-phase serum sample (weeks after fever onset) (n + 1 vs. n, n = 3 through 15 wk)	–	–	0.449 + 0.198	0.026
(Duration of illness) ×(Time of convalescent-phase serum sample)	–	–	–0.005 + 0.0024	0.037
(Time of convalescent-phase serum sample)^2^	–	–	–0.025 + 0.012	0.042

*Logistic model: age, with every additional year of age, the odds of seropositivity is 0.97 (odds ratio, OR) (see Figure 1); sex, the odds for women to be seropositive is 1.24 (OR) when compared with men; infectious source, the odds of patients with known infection source to be seropositive is 15.6 times that of the patients without known source of infection; duration of illness, for every additional day of illness, the odds of seropositivity increases by 1.08. †Linear mixed model: log_2_ (neutralizing antibody titer) = β_0_ + β_1_ (age) – β_1_ (sex) + β_3_ (infection source) + β_4_ (duration of illness) + β_5_ (time of convalescent-phase serum sample) –β_6_ (duration of illness ×time of convalescent-phase serum sample) –β_7_ (time of convalescent-phase serum sample)^2^. In results above, the model estimates are based on log_2_ (titers), to which the time of convalescent-phase serum collection (in weeks postonset of illness, starting from week 3) contributed in 3 terms; the antibody rise follows the first order of weeks postonset, and decay follows the second order of weeks postonset and an interactive term between duration of illness and weeks postonset.

### Antibody and Duration of Illness

The total number of serum specimens collected from each patient ranged from 1 to 4, including >1 convalescent-phase serum sample collected after week 4. Of the 247 seropositive SARS patients, 217 (87.8%) of the patients were seropositive by week 3; 27 (17.4%) of 155 patients who were seropositive within the first 2 weeks of illness and are called early responders hereafter. On rare occasions (1.21%, 3/247), seroconversion occurred after week 6 of symptoms onset.

**Figure 2:**
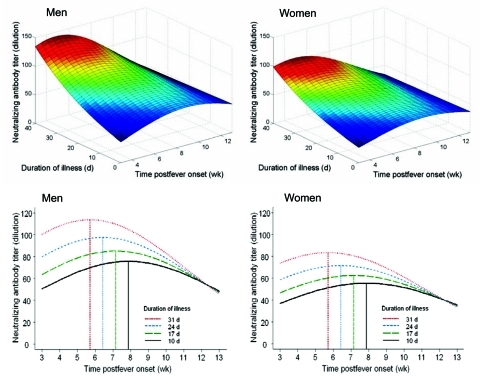
A) Perspective surfaces of neutralizing antibody titer (dilution) based on the fitted linear mixed model in Table 5. The median age was 36 years for men (left panel) and for women (right panel). B) Cross-sectional curves of neutralizing antibody titer (dilution) extracted from panel A with duration of illness set at 10, 17, 24, and 31 days, respectively, for men (left panel) and women (right panel); the vertical lines mark peak titer times.

**Figure 3:**
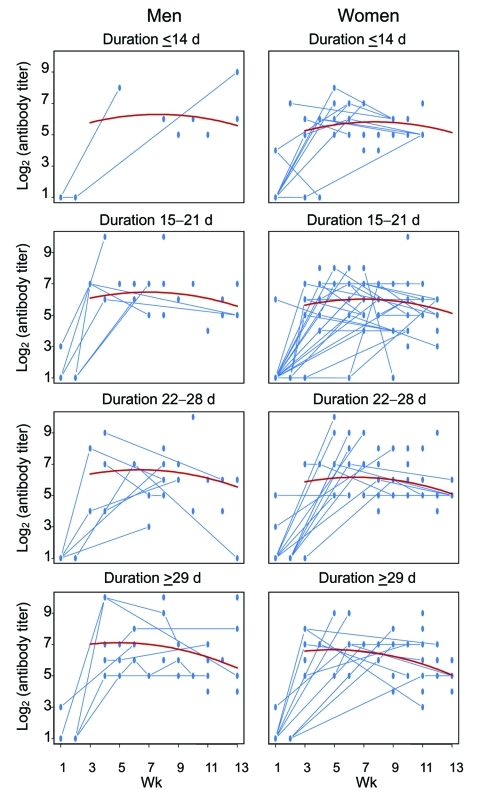
Scatterplot of antibody titers of the 247 seropositive study participants (titers of the same participant measured at different times are connected); superimposed is the fitted mean curve (in red) of log2 (antibody titer) between weeks 3 and 13 postinfection based on the linear mixed model by severity (duration of illness) and sex at the median age of 36 years. Each dot represents >1 titer; no distinction is made between single values and those with >1 value.

Because of the differences in the distribution of age and sex among patient groups and the differences in the number and timing of specimens collected for antibody measurement, we used regression-based modeling approach to examine these factors simultaneously and tried to analyze the relationship between their potential interactions and antibody titers (Table 5, Figure 2A). This model was based on the neutralizing antibody titer of the 312 convalescent-phase serum assays, representing 194 patients who had had 1 convalescent-phase serum sample collected between weeks 3 to 12 after onset of fever, 41 patients who had 2 convalescent-phase samples, and 12 patients who had 3. The number of serum specimens collected ranged from 21 to 43 per week from week 4 of illness through week 12. The model suggested that neutralizing antibody rose and diminished during the follow-up period between weeks 3 and 13 after onset of illness (p = 0.026 for linear term and p = 0.042 for quadratic term); the estimated half life was ≈6.4 weeks. Patients with a more protracted clinical course tended to have a higher antibody titer than patients with a shorter clinical course (p = 0.008). Antibody in patients with more severe clinical courses tended to decay at a faster rate than in patients with shorter clinical course (the interaction between duration of illness and time of serum collection, p = 0.037). This pattern of decay followed a half-life of ≈6.4 weeks after reaching the peak, which occurred between weeks 5 and 8 after infection (Figure 2B). The time that the blood was collected for each patient was examined, and an equally dispersed pattern of blood collection was found in all clinical groups (Figure 3).

Of the 53 patients who had a second convalescent-phase specimen collected after 6 weeks, 16 (31.2%) of 53 showed a 4-fold (2 dilution) drop by week 12 postinfection. Three patients had a negative seroconversion during the same period. Conversely, a measurable neutralizing antibody persisted in 12 (85.7%) of 14 patients who had been followed up between weeks 13 and 16. To better examine the dynamics of the antibody profile, cross-sectional views of Figure 2A were extracted (Figure 2B). The model suggested that antibody response was higher and occurred earlier in patients with a more severe clinical course than in those with a shorter clinical course.

### Early Responders and Deaths

In the model, patients with a more severe clinical course had earlier and higher antibody responses; we then examined the death rate of the early responders (Table 6). These early responders had a significantly higher mortality rate (29.6% vs. 7.8%) than others who did not undergo seroconversion until week 3 of illness or later (p = 0.004, χ^2^ test). These early responders also tended to die early during the acute phase: 6 of 8 died during the first 2 weeks of illness, and the other 2 died on days 15 and 17 of illness, respectively (Fisher exact test, p = 0.028). Of the 10 patients who died and who seroconverted after the second week of symptom onset, only one died during the first 2 weeks of illness (Wilcoxon rank sum, p = 0.007). Among the 27 early seroresponders, the antibody titer of those who died (n = 8) (median titer = 48) was not significantly higher than that of those who survived (median titer = 32) (Wilcoxon rank sum, p = 0.79). However, the early seroresponders were significantly older (mean age 43.7 years) than the 128 case-patients who seroconverted after week 2 of illness (mean age 37.3 years) (Wilcoxon rank sum, p = 0.028); i.e., older patients were more likely to be early responders, 60% of patients >60 years of age versus 16.3% of patients <60 years (Fisher exact test, p = 0.004).

**Table 6:** Comparison of early seroresponders and other SARS patients, by death rate, time of death, and age*

Factor	Early seroresponders†		OR	p value‡
	Yes, n (%)	No, n (%)		
Died				
Yes	29.6 (8)	7.8 (10)	4.97	0.004
No	70.4 (19)	92.2 (118)	1.00	
Died within 2 wk				
Yes	75.0 (6)	10.0 (1)		0.013
No	25.0 (2)	90.0 (9)		
Age (y)				
>60 (n = 14)	42.9 (6)	57.1 (8)		0.01
<60 (n = 141)	14.9 (21)	85.1 (120)		

*SARS, severe acute respiratory syndrome; OR, odds ratio. †Early seroresponders are SARS patients who were seropositive for SARS neutralizing antibody during the first 2 weeks of illness. ‡p value based on Fisher exact test.

## Discussion

Neutralizing antibody plays an integral role in immunoprotection from viral diseases, and serologic tests are important to their diagnosis. This report relates SARS neutralizing antibody profiles to clinical outcomes. The lack of a readily available, well-characterized diagnostic assay that could be used as a standard and the additional lack of a well-established typical clinical description that encompasses all clinical syndromes are intrinsic difficulties of working with a new infectious disease. Therefore, analysis of the interrelation between clinical, epidemiologic, and laboratory data might provide further insight into these elements. Results of our analysis, although they passed a certain level of statistical scrutiny, should be interpreted with caution.

### Antibody Titer and Seronegativity

The 32 seronegative SARS patients whose diagnoses were based on positive RT-PCR results of nasopharyngeal swab specimens warrant further discussion concerning whether they were indeed SARS patients or were merely misdiagnosed by the false-positive RT-PCR of SARS-CoV. The false-positive RT-PCR is most commonly due to cross-contamination, which pertains to the nature and quality of a laboratory procedure and should be independent of patient's profile. However, we found that seronegative patients were more likely to have a short duration of illness and no clear source of infection. Lack of specificity of the test is another reason for having a false-positive RT-PCR, but none of the commercial tests we used have been reported to have nonspecific cross-reactivity with other known pathogens. Furthermore, after May 1, SARS diagnosis required positive results of >2 specimens collected at different time or from different sites, or tested by >1 RT-PCR method (Centers for Disease Control and Prevention, Atlanta, GA, USA; Roche Diagnostics GmbH, Mannheim, Germany; Artus GmbH, Hamburg, Germany) if only 1 specimen was available. Therefore, a specimen that yields false-positive results with 2 different test methods is deemed unlikely.

Alternatively, these 32 patients were indeed SARS patients, but the negative neutralizing antibody reading was due to patent's low antibody level in combination with the low sensitivity of the antibody test. The sensitivity of our neutralizing assay is comparable to that of ELISA (*9*), but the possibility that our assay had a low sensitivity remains because the neutralizing antibody test is based on the reading of a complete inhibition of cytopathic effect. Thus the absolute titer is expected to be lower than the results, based on reading of 50% inhibition. The neutralizing antibody of SARS patients has been reported in only 1 other study, in which a pseudovirus containing the S protein of SARS-CoV was used; antibody titers were found to be low (*19*). A low antibody response may be associated with a primary infection of SARS-CoV, as seen with primary infection of respiratory syncytial virus (RSV) (*20*), and infection through the respiratory tract was shown to stimulate a less vigorous immune response than infection by an invasive intravenous rejection of RSV (*21*). Furthermore, a robust humoral immune response requires antigen in sufficient doses through a proper route; this fact has been demonstrated in vaccine studies, including research on several live vaccines (*22**-**24*). The lack of detectable antibody among patients without history of contact with a known SARS patient might be associated with a low inoculum of the virus because of incidental exposures, in contrast to patients who acquired SARS in hospitals under circumstances assumed to have a high virus density. When systematically screened during the SARS outbreak, some healthcare workers and public health personnel who had a history of direct contact with patients were shown to harbor nasopharyngeal SARS-CoV. Subsequently, however, they did not show seroconversion (Y.-T. Lu et al., unpub. data), which raises the possibility of asymptomatic mucosal epithelial colonization by SARS-CoV. Our seronegative patients with mild symptoms might fit into the spectrum between the seronegative asymptomatic colonizers and the severe SARS patients with high neutralizing antibody response. All considerations appear to favor the possibility that seronegative patients indeed had acquired SARS. Since the natural reservoir of SARS-CoV has not been clearly identified, and reintroduction of SARS-CoV to humans is possible, the short duration of having detectable antibody should be considered when a vaccine against SARS-CoV is developed.

### Severity and Pathogenesis

The clinical course of SARS patients with severe infection is described as follows: pulmonary functions worsen during week 2 of illness (*5**,**25**-**27*), while the virus load in the airway decreases (*5*), and patients with mild disease would begin to stabilize clinically. Those in whom ARDS later develops usually show pulmonary decompensation during week 2. Severity was intensified by a slower and prolonged recovery with complications of pulmonary fibrosis occurring in week 3 in some patients (*16*). Results of a high-resolution computed tomographic scan in follow-up of SARS patients corroborates this observation by showing a high correlation between bilateral fibrotic lung changes and clinical severity (*28*). Findings of these studies, in conjunction with clinical study on cytokines during the acute phase (*29**-**31*), suggest that activation of Th1 cell–mediated immunity and a hyperinnate inflammatory response, rather than direct damages from uncontrolled virus growth, are responsible for the pathogenic process in severe infection (*5*). In previous vaccine studies, immunization conditions that could induce a stronger activation of Th1 response would concurrently result in a higher antibody response (*24**,**32*). Thus, the high antibody response and a strong cell-mediated Th1 response may reasonably be understood as concurrent events, and the latter may be causally related to a severe clinical course of SARS. Neutralizing antibody is unlikely to be causally related to the pathogenesis of SARS because treating SARS patients with convalescent-phase serum collected from patients who had recovered from SARS showed no adverse effect and probably had beneficial effects (*33*). Thus, for all the reasons stated above, our finding that high neutralizing antibody correlating with clinical severity should not be interpreted to mean that neutralizing antibody is harmful.

### Death Rate and Early Responders

Having a detectable neutralizing antibody during the first 2 weeks of illness, in our analysis, coincides with a high and an early SARS mortality rate. The basis for early antibody response is not apparent, but 1 possibility is the priming effect of a previous non–SARS-CoV infection. Indeed, antibody against SARS-CoV has been shown to cross-react with human coronavirus 229E (*2*). The finding that early responders are older than other SARS patients is in agreement with the priming effect since cumulative infection rate increases with increasing age. The priming effect of a previous viral infection can induce cross-reactive but nonneutralizing antibody, as well as neutralizing antibody to SARS. Furthermore, the nonneutralizing antibodies are known to facilitate viral infection, termed antibody-dependent enhancement (ADE), which is the pathogenic basis of feline infectious peritonitis virus (FIPV, a type II coronavirus), dengue hemorrhagic fever, and other viruses (*34**–**36**,**37*). In the case of FIPV, ADE can occur even with neutralizing antibody (*38*). However, this type of ADE resulted directly from neutralizing antibody is unlikely to occur with SARS-CoV infection because a number of SARS patients have been treated with convalescent-phase serum of SARS patients and show no adverse effect (*33*).

The hypothesis that the early responders may have experienced a priming effect could be verified by demonstrating that a significantly higher proportion of early responders than other SARS patients possess antibody against non-SARS coronavirus during the acute phase. Early death occurring within the first 2 weeks of illness is also associated with high nasopharyngeal virus load among a subset of SARS patients with information on nasopharyngeal virus load (J.-Y. Yang et al., unpub. data). Unfortunately, the number of early responders for whom information on virus load was available was too few to yield a meaningful statistical analysis on whether high virus load is correlated with an early humoral response. While antibody induced by a variety of SARS-CoV antigen preparations protects against SARS-CoV infection in mice and ferrets (*39**,**40*), these animals do not develop clinical symptoms resembling that of SARS-CoV infection in humans and thus are not models of pathogenesis. Since ADE can occur through a number of mechanisms and is not completely understood, clinical trials of vaccine against SARS-CoV should be conducted with caution.

In summary, SARS neutralizing antibody level is positively correlated with clinical severity, and in a portion of the patients with mild infection, a detectable neutralizing antibody response may not develop. All published clinical and immunologic data on SARS patients suggest that a strong cell-mediated Th1 response is causally related to a severe clinical outcome, whereas high neutralizing antibody is probably a concurrent event of a strong Th1 activation. Early neutralizing antibody responders are more likely to be older, to have a higher case-fatality rate, and to survive for a shorter time. These observations, if corroborated with further analysis of data collected in other countries, should raise the concerns of possible ADE in the pathogenesis of SARS-CoV infection in humans and should be considered in the process of vaccine development.
